# Multilevel approaches to increase fruit and vegetable intake in low-income housing communities: final results of the ‘Live Well, Viva Bien’ cluster-randomized trial

**DOI:** 10.1186/s12966-018-0704-2

**Published:** 2018-08-20

**Authors:** Kim M. Gans, Patricia Markham Risica, Akilah Dulin Keita, Laura Dionne, Jennifer Mello, Kristen Cooksey Stowers, George Papandonatos, Shannon Whittaker, Gemma Gorham

**Affiliations:** 1 Department of Human Development and Family Studies, University of Connecticut, Storrs, USA; 2 University of Connecticut Intitute for Collaboration in Health, Interventions and Policy, Storrs, USA; 3 Center for Health Equity Research, Brown University School of Public Health, Providence, USA; 4 Deartment of Behavioral and Social Science, Brown University School of Public Health, Providence, USA; 5 University of Connecticut Rudd Center for Food Policy and Obesity, Hartford, USA; 6 Department of Statistical Scieces, Brown University School of Public Health, Providence, USA; 7 Yale University School of Public Health, New Haven, USA

**Keywords:** Diet, Nutrition, food access, Fruit and vegetable, Farmer’s market, Mobile market, Nutrition education, housing, community, food environment

## Abstract

**Background:**

Fruit and vegetable (F&V) intake can reduce risks for chronic disease, but is much lower than recommended amounts in most Western populations, especially for those with low income levels. Rigorous research is needed on practical, cost-effective interventions that address environmental as well as personal determinants of F&V intake. This paper presents the results of a cluster randomized controlled trial evaluating the efficacy of ‘Live Well, Viva Bien’ (LWVB), a multicomponent intervention that included discount, mobile fresh F&V markets in conjunction with nutrition education.

**Methods:**

Fifteen subsidized housing sites in Providence County, Rhode Island (8 intervention and 7 control sites) were randomized using a random number generator. Of these, nine housed elderly and/or disabled residents and six housed families. A total of 1597 adult housing site residents (treatment *n* = 837; control *n* = 760) were enrolled (73% women, 54% Hispanic, 17% black, Mean age 54 years). A year-long multicomponent intervention including mobile F&V markets plus nutrition education (e.g. campaigns, DVDs, newsletters, recipes, and chef demonstrations) was implemented at intervention sites. Physical activity and stress interventions were implemented at control sites. Follow-up occurred at 6 and 12 months. The main outcome measure was F&V consumption measured by National Cancer Institute’s ‘Eating at America’s Table All Day Screener’.

**Results:**

From baseline to 12 months, the intervention group increased total F&V intake by 0.44 cups with the control group decreasing intake by 0.08 cups (*p* < .02). Results also showed an increased frequency of F&V eating behaviors compared to the control group (*p* < .01). There was a clear dose response effect of the F&V markets with participants who reported attending all or most of the markets increasing F&V intake by 2.1 cups and 0.86 cups, respectively compared with less than half cup increases for lower levels of market attendance (*p* < .05). Use of the DVDs, recipes and taste-testings was also associated with greater increases in F&V intake; however, use of other educational components was not.

**Conclusions:**

LWVB is the first cluster, randomized controlled trial to demonstrate the efficacy of year-round F&V markets on improving F&V intake for low-income adults, which provides an evidence-base to bolster the mission of mobile produce markets. Further, the results more broadly support investment in environmental changes to alleviate disparities in F&V consumption and diet-related health inequities.

**Trial registration number:**

Clinicatrials.gov registration number: NCT02669472

## Background

Fruit and vegetable (F&V) intake can promote health, prevent obesity, and lower risks for hypertension, coronary heart disease, stroke, type 2 diabetes, some cancers and all-cause mortality [1–6].A comparative risk assessment of the global burden of disease identified diets low in F&V to be one of the five leading risk factors worldwide [7]. Despite potential benefits, F&V consumption is much lower than recommended amounts in most Western populations [8]. Although dietary recommendations vary among countries, most are in line with the World Health Organization’s recommendation to consume a daily minimum of 400 g of F&V, or the equivalent of five servings of F&V per day [9]. In the United States [10, 11], 37.7 and 22.6% of U.S. adults report consuming F&V less than once per day; only 18% meet the dietary guidelines for fruits and only 13% meet guidelines for vegetables [12]. Groups at greater risk for low F&V consumption include those of low-income or low educational status [12]. These socioeconomic status (SES) disparities in F&V consumption are partly attributable to the food environment in low-income neighborhoods, where residents often have limited access to affordable, healthful food [13]. Two of the most common reported barriers to F&V consumption are, in fact, high cost and limited access [14, 15].

Farmer’s markets and mobile F&V markets have emerged as innovative and promising approaches for increasing access to healthful food [16]. Although such markets demonstrate potential for improving dietary intake, there have been no randomized trials studying their efficacy to date. Moreover, as Dibsdall, et al. found, access and affordability are only two of the factors contributing to low F&V consumption for low SES adults; personal determinants need to be addressed as well [17]. The most common personal factors are hectic lifestyles (leaving little time for shopping and cooking), taste preferences, negative attitudes and perceived norms regarding healthy eating, and lack of knowledge, skills, self-efficacy and social support [18]. Thus, research is needed on practical, cost-effective interventions that not only improve F&V access and affordability, but also address these barriers. Also, the majority of studies to date have focused mainly on individual level barriers to F&V consumption rather than on environmental level barriers. The purpose of this paper is to present the final results of the ‘Live Well, Viva Bien’ (LWVB) trial, which was designed to fill these research gaps by addressing both personal and environmental determinants to increase F&V consumption for low-income populations. Results from the current study can inform the development of local partnerships (e.g, between produce markets and hospitals or public housing agencies) [19], institutional policies (e.g., at the food-bank level) as well as public policies shaping access to federal food assistance aiming to improve access to F&V among low-income populations and other marginalized populations.

## Methods

### Overview

‘Live Well, Viva Bien’ (LWVB) was a cluster, randomized controlled trial designed to evaluate the efficacy of a multicomponent intervention that included discount, mobile fresh F&V markets in conjunction with a nutrition education intervention [20]. All study activities occurred at 15 subsidized housing complexes in Providence County, Rhode Island with 8 sites receiving the F&V intervention and 7 sites receiving a comparison intervention. Pre-intervention focus groups were conducted with housing complex residents (from non-study sites) to inform intervention development. Adult residents from each housing site were recruited for the evaluation cohort prior to site randomization. The multicomponent intervention lasted 1 year and included baseline, 6-month and 12-month evaluation surveys as well as extensive quantitative and qualitative process evaluation throughout the course of the study. Participants were given a $30 gift card incentive upon completion of each of the three surveys ($90 total). All study protocols were approved by the Brown University Institutional Review Board and all participants provided informed consent. Details of the intervention components and trial protocols are presented elsewhere [20].

### Recruitment of housing sites

The Providence, Pawtucket and Woonsocket Housing Authorities assisted with the selection and recruitment of the subsidized housing complexes that participated in the study. To be eligible, the housing complex needed to have: at least 190 units; a relatively low turnover rate (< 20%); a community room or center; a willingness to be randomized into one of the two experimental conditions, and support for the study activities for the duration of the study. In addition, at least 90% of the residents needed to be able to speak and read either English or Spanish. Housing site pairs were matched by number of units, type of site (family or elderly/disabled) and race/ethnicity of residents. Of the 15 sites ultimately recruited, 9 were elderly/disabled sites and 6 were family sites. A Resident Assistant was hired from each participating site to assist with recruitment and intervention activities.

### Participant recruitment

Though intervention activities were open to all housing complex residents approximately 100 residents per site were recruited to participate in an evaluation cohort. Recruitment began with an onsite, recruitment event prior to which posters were displayed throughout the housing complex and informational hangers were placed on each apartment door. Research staff also knocked on apartment doors to invite residents to participate. Interested residents then met with bilingual Brown research staff in the community room of the housing complex where they were screened for eligibility. To participate in the evaluation cohort, residents needed to: be 18 years of age or older; be full-time residents of the housing complex; shop for their household’s food at least half of the time; not have any major medical conditions that would prevent them from participating in study activities or events; not be planning to move in the next year; be able to read and understand either English or Spanish; and have access to a Digital Video Disk (DVD) player (or computer that could play DVDs).

### Baseline surveys and randomization

Baseline surveys were then conducted with eligible participants either in person or via computer-assisted telephone interviews. After the baseline surveys were completed at each pair of sites, the project data manager randomly assigned the sites to either the intervention or control group using a random number generating function in Excel. The study enrolled a total of 7 sites in the control group and 8 sites in the intervention group, which included one pilot site. If housing residents were ineligible or did not choose to be in the evaluation cohort, they were still invited to participate in the intervention activities. As randomization was at the site level, participants were not blinded to the intervention condition; however, Brown evaluation staff were blinded to the group assignment of the sites.

## Intervention

### Formative research

Ten focus groups (eight exploratory and two confirmatory) were conducted with 79 low-income, racially and ethnically diverse residents at three, non-participating housing sites to inform the development of recruitment, intervention and evaluation materials [20].

### Intervention framework

**Fig. 1:**
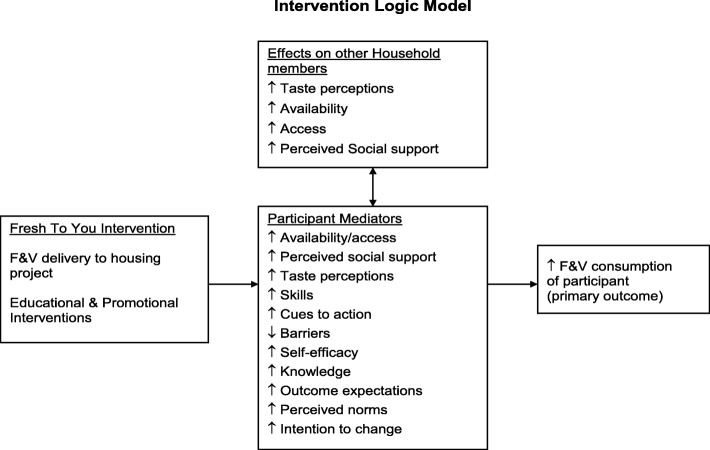
Intervention Logic model of the Live Well/Viva Bien study

The multicomponent LWVB intervention was based on a social ecological model, which recognizes that behavior is affected by multiple levels of influence and that an intervention will be most effective when it targets changes in multiple levels or domains [21–23]. The multi-level LWVB intervention operated within the intrapersonal/individual, interpersonal (social), and environmental domains. The theoretical framework for the LWVB intervention was the Social Cognitive Theory (SCT), which defines behavior as a triadic, dynamic, and reciprocal interaction of personal factors, behavior and the environment [24–26]. See the Logic Model in Fig. 1 and previous work for further details about how theory informed the intervention [20].

### Intervention components

The 12-month intervention featured discount, fresh F&V markets called ‘Fresh to You’ (FTY), as well as a multicomponent, educational intervention. F&V prices were set each day by the market coordinator, who attempted to keep the prices at or below the retail prices at local supermarkets by looking at online grocery store produce prices. The intervention began with a highly-publicized ‘Kick-Off’ event, during which we brought the first FTY market to each intervention site, along with chef-run cooking demonstrations/taste-testing events, recipes and detailed information about upcoming intervention activities. Regularly scheduled, discount, fresh F&V markets continued to be held at each intervention site for a one-year period [20, 27]. Between 50 and 70 different, local and non-local produce items (including staples, seasonal items, culturally-desired ethnic produce and exotic produce) were sold at the markets at or below retail (supermarket) prices. The types of F&V sold at the market were informed by the pre-study focus groups as well as by customer comments and requests throughout the study. These requests were forwarded to the market coordinator, who then made sure those products were available at future markets at that site. The markets lasted 2 hours and were always held *indoors* at the senior and disabled housing complexes and during inclement weather at family housing complexes. In good weather, the markets were held *outdoors* at the family housing complexes in a car trailer that had been retrofitted to serve as a mobile market [20]. We originally planned to bring weekly markets to each intervention site. However, limited resident participation in the markets during the third and fourth weeks of the month led us to reduce the frequency of the markets to the first 2 weeks of each month. It became clear early in the study that residents were much more likely to shop soon after receiving their monthly Supplemental Nutrition Assistance Program (SNAP) benefits on the 1st of each month.

Before the first market was held, an educational/informational packet was delivered to all intervention housing complex residents that included a large, reusable FTY shopping bag containing a binder that included an overview of the intervention, the first month’s newsletter, three educational DVDs, 48 recipe cards and three-hole binder sleeves to store the remaining newsletters they would receive over the course of the intervention. All materials were provided in English or Spanish. A brief description of each motivational/educational intervention component follows. See Gans et al. [20] for more detail.

The intervention included 2 six-week educational/motivational campaigns. The first campaign (*‘Just Add 2’*) began soon after the baseline surveys were completed and was designed to increase participants’ daily F&V consumption by two servings. The second campaign (*‘Color Your Plate’)* focused on increasing the variety of F&V that participants ate and began soon after the 6-month evaluation surveys were completed. Both campaigns included full-color booklets with goal-setting activities, educational and motivational content, and F&V trackers. A midpoint and final event were held at each intervention site that included chef-led cooking demonstrations, taste-testing events and raffles with prizes.

Three, 20-min DVDs and 48 corresponding recipe cards were developed and distributed to all intervention housing complex residents to support and encourage increased F&V intake. The DVDs included FTY market information; cooking demonstrations; success stories and descriptions of health benefits associated with F&V. Each month, a two-page, full-color newsletter was delivered to the door of each intervention housing complex resident. The newsletters highlighted the produce in season that month, its key nutrients, health benefits along with recipes and information regarding how to choose, prepare, store and save time and money purchasing and preparing F&V. Six times during the year, a chef presented cooking demonstrations and taste-testing events at each intervention housing complex and provided attendees with corresponding English/Spanish recipe cards.

### Comparison/control intervention

Two, six-week, non-nutritional, educational and motivational campaigns were provided to residents of the 7 comparison group housing complexes. The *‘Take 10!’ Campaign* aimed to increase residents’ daily physical activity by 10 min per day. The *‘Stress Less’ Campaign* aimed to help participants reduce stress by adding stress-reduction activities into their daily routines. These comparison group campaigns included the same types of information and activities as the intervention group campaigns as well as raffles with prizes. Campaign participants also received a free, 6-week membership to the YMCA.

## Process evaluation

Detailed FTY market sales data (e.g. total sales, # of shoppers, items purchased and tender types) were captured by the FTY markets’ point-of-sale cashiering system. Brown research staff recorded the number of residents who participated in the campaigns and other intervention-related activities. The 6- and 12-month follow-up surveys also included process evaluation questions regarding participation in and perceptions of each component of the intervention as well as open-ended questions regarding what participants liked/disliked about the program, what they learned from the program, and how the program could be improved. A subset of raw responses to the open-ended questions was reviewed by the investigators and research staff, who then created coding categories for the responses. Two staff members independently coded the responses into themes and met regularly with the data manager to decide how to handle responses that were coded differently. Additional coding categories were added as new themes emerged.

## Effect evaluation

Baseline, mid-term and 12-month follow-up surveys were conducted by Brown research staff, either in-person at the housing complex, or by phone via computer-assisted telephone interviewing. All data that were collected in person were first reviewed for completeness and then entered into the online computer database. Baseline surveys began in June 2011 for the pilot site and ended in August 2013 for the last pair of sites; 12-month follow-up surveys were completed for the last pair of sites in October 2014.

### Outcome measures

The study’s primary outcome was F&V intake measured using the 18 item National Cancer Institute (NCI) ‘Eating at America’s Table All Day Screener’. [28] The Screener queries 18 F&V consumed over the past month. Participants were asked to report the frequency (from never to 5 or more times per day) of F&V they ate last month and serving size (from less than ½ cup to more than 1½ cups) for each F&V group in the survey. Response options were standardized to a daily cup serving and then total F&V consumption was calculated by summing the products of each F&V group. The responses to the frequency questions were recoded to daily averages based on standard NCI methods [28]. Screener data was recoded to examine changes in intake of fruits omitting juice, vegetables omitting French fries, and F&V together omitting juice and French fries.

Additionally, F&V practices were assessed in a series of eleven questions adapted from previous food habits questionnaires [29–31]. These questions included how often in the past few months participants: ate fruit at breakfast; added vegetables to breakfast dishes; ate more than one type of fruit per day; ate more than one type of vegetable per day; ate a lettuce-based salad or vegetable at lunch; ate fruit at lunch; ate a lettuce-based salad or vegetable at dinner; ate two or more different vegetables or a vegetable and a salad at dinner; added vegetables to other foods or dishes; ate a fruit or vegetable as a snack in-between meals; and ate just fruit as dessert instead of a rich dessert. Each question had five levels of response (always, often, sometimes, rarely or never). Responses were scored so that higher scores were indicative of higher F&V intake behaviors and then the sum of all scores was calculated as the total Fruit and Vegetable Habits Questionnaire (FVHQ) score.

### Demographic measures

Demographic variables were categorized as follows: age (18–39, 40–59, 60+); gender (male vs. female); marital status (single, married, divorced, separated, widowed, other); race (White, Black or African American, mixed race, or other); birth country (United States vs other), years lived in US (0–5, 6–10, 11–15, 16–19, 20+); Hispanic (yes/no); Hispanic culture (Dominican, Puerto Rican, and other, which included Columbian, Guatemalan, Mexican, Cape Verdean, among other groups); country of origin (US, other country); language(s) spoken at home (English only, Spanish only, Both, more English than Spanish, Both equal amounts of time, Both more Spanish than English, or Other language); employment status (full time, part time, unemployed, disabled, retired, student/homemaker); education (First to 9th grade, Grades 10–12, Vocational/Technical School or Some college; BA Degree/Post graduate); household income (Less than $6000, $6000–$11,999, $12,000–$17,999, $18,000+); number of adults and children living in household [1–9]; and participation in food assistance programs (Any of the following programs: WIC, SNAP (Food Stamps), Emergency Food Program, Summer Food Service Program, Senior Citizen Meal Program, Meals on Wheels Program) (yes/no). At the site level, we also looked at type of housing site (elderly/disabled vs. family).

### Data analysis

To compare demographic characteristics by experimental group (control v. intervention), chi square tests were used for categorical variables and analysis of variance was used for continuous variables. To examine changes in F&V intake, linear mixed effects models (SAS PROC MIXED) were generated. The model included fixed effects for time and random intercepts for housing site and participants nested within the housing sites, and does not require imputation for missing data assuming that data are missing-at-random (MAR) [32]. Robust standard errors were used in order to correct for heteroscedasticity. Site level intra-class correlation was calculated for residents of the same site across time, as well as between residents of the same site. See Appendix. Because it was possible for participants to move from one site to another during the study, we asked residents and omitted data from those that changed sites. Month 6 follow-up data was omitted for participants (*n* = 17) who moved anywhere outside of their original housing site before exposure to 4 months of intervention (and they were excluded from the month 12 evaluation). Month 12 follow-up data was omitted for participants (*n* = 7) who moved anywhere outside of their original housing site before exposure to 8 months of intervention.

Dose variable distributions were explored. Given the broad distribution of participation, market participation was assessed including all response levels (all, most, some, few, none). For recipe use, the response of, “none,” was collapsed with the response, “I didn’t get recipes.” Variables for newsletter reading was collapsed at the middle category comparing those who read at least half of the newsletter compared with less than half of the newsletter. Lower use compelled collapsing variables for DVD watching, participation in Taste Tests and Campaigns to watching or participation in one or more with watching or participation in none of the DVD/taste tests/campaigns. T-tests for each outcome of fruit, vegetable or F&V consumption were assessed by each dose variable. All analyses were performed using SAS version 9.4 (SAS Institute, Cary, NC).

## Results

**Fig. 2:**
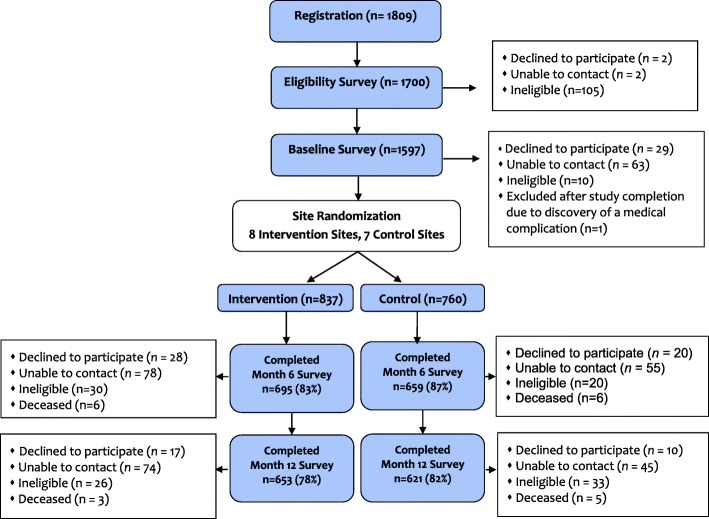
Consort diagram. Recruitment, enrollment and retention of evaluation cohort participants

A total of 1597 housing complex residents were enrolled in the evaluation cohort (Fig. 2), which represents 88% of those who initially registered. Just over half (56%) of the participants were recruited from elderly/disabled housing complexes and 44% were recruited from family housing complexes. A total of 837 participants were enrolled at the intervention sites and 760 at the control sites. Follow-up evaluation was conducted with 695 intervention group participants (83%) and 659 comparison group participants (87%) at 6 months (*p* < .05) and with 653 intervention group participants (78%) and 621 comparison group participants (82%) at 12 months (*p* < .06).

Participants enrolled (Table 1) were mostly women (73%) with a mean age of 54 years. The largest marital status category was single (43%); only 14% were married. Less than half (48%) of participants identified their race as White; 17% as Black, 20% as Mixed Race and smaller percentages of other racial groups. The majority of participants were Hispanic (54%) of which 45% were Dominican and 44% Puerto Rican, with an additional 11% from other cultural groups. Of those not born in the US, which comprised about half of the study sample, 46% reported having been in the country more than 20 years. Regarding language, 41% spoke only English at home, with 19% speaking only Spanish and 33% speaking both languages. The largest group of participants (33%) reported their employment status as “disabled,” while 21% reported that they were unemployed; 21% retired, and only 15% working full or part time. In terms of education, 35% said they had less than a 10th grade education; 46% had a 10th -12th grade education and only 4% earned a college degree. Most participants (68%) reported a household income of less than $12,000 per year including 16% who reported less than $6000 per year. More than half (55%) reported living alone.

**Table 1:** Demographics of Study Sample Consisting of Adults aged 18+, by Experimental Group (*N* = 1597)

Variable	Category	All % (n)	Intervention % (n)	Control % (n)	*p*-value
Site	Elderly	56.5 (903)	57.2 (479)	55.8 (424)	0.56
	Family	43.5 (694)	42.8 (358)	44.2 (336)	
Gender	Male	26.6 (425)	25.7 (215)	27.6 (210)	0.38
	Female	73.4 (1172)	74.3 (622)	72.4 (550)	
Age category	18–39	24.5 (392)	24.1 (202)	25 (190)	0.01*
	40–59	33.5 (535)	36.7 (307)	30 (228)	
	60+	42 (670)	39.2 (328)	45 (342)	
Mean age		53.7 (1597)	53.5 (837)	53.9 (760)	0.68
Marital status	Single	42.9 (683)	45.6 (380)	40 (303)	0.07
	Married	14.4 (229)	15.1 (126)	13.6 (103)	
	Divorced	17.7 (281)	16.6 (138)	18.9 (143)	
	Separated	10.2 (162)	9.1 (76)	11.3 (86)	
	Widowed	13.3 (211)	12.5 (104)	14.1 (107)	
	Other, please describe	1.6 (25)	1.1 (9)	2.1 (16)	
Race	White	48 (715)	49.5 (383)	46.2 (332)	0.24
	Black	17.3 (258)	18.1 (140)	16.4 (118)	
	Mixed	19.5 (291)	18.4 (142)	20.8 (149)	
	Other	15.2 (227)	14 (108)	16.6 (119)	
Hispanic	Yes	53.9 (857)	55.2 (460)	52.4 (397)	0.26
	No	46.1 (734)	44.8 (373)	47.6 (361)	
Hispanic Culture	Dominican	44.5 (379)	44.2 (202)	44.8 (177)	0.25
	Puerto Rican	44.4 (378)	46.2 (211)	42.3 (167)	
	Other	11.2 (95)	9.6 (44)	12.9 (51)	
What country were you born in	United States	50.2 (799)	49 (410)	51.4 (389)	0.35
	Other Country	49.8 (794)	51 (426)	48.6 (368)	
How many years have you lived in the US	0–5 Years	11.7 (93)	11.3 (48)	12.1 (45)	0.99
	6–10 Years	14.4 (115)	14.6 (62)	14.3 (53)	
	11–15 Years	14.4 (115)	14.1 (60)	14.8 (55)	
	16–19 Years	13.3 (106)	13.2 (56)	13.5 (50)	
	20 Years or more	46.1 (367)	46.8 (199)	45.3 (168)	
What languages are spoken in your home	English only	41.1 (657)	40.4 (338)	42 (319)	0.37
	Spanish only	19 (303)	19.1 (160)	18.8 (143)	
	Both, more English than Spanish	9.6 (153)	9 (75)	10.3 (78)	
	Both, equal amounts of time	8 (127)	8.8 (74)	7 (53)	
	Both, more Spanish than English	15.5 (248)	16.6 (139)	14.3 (109)	
	Other language, please describe	6.8 (109)	6.1 (51)	7.6 (58)	
Employment Status	Full time	5.2 (83)	5.5 (46)	4.9 (37)	0.08
	Part time	10.4 (165)	8.6 (72)	12.3 (93)	
	Unemployed	21.4 (341)	21.4 (179)	21.4 (162)	
	Disabled	33.1 (527)	35.2 (294)	30.8 (233)	
	Retired	20.6 (327)	19.3 (161)	22 (166)	
	Student/Homemaker	9.3 (148)	9.9 (83)	8.6 (65)	
Education	First - 9th grade	34.9 (554)	35.2 (293)	34.6 (261)	0.99
	Grades 10–12	45.7 (725)	45.7 (380)	45.8 (345)	
	Vocational/Tech/Some college	15.3 (243)	15 (125)	15.6 (118)	
	BA degree/Post grad	4 (64)	4.1 (34)	4 (30)	
Household Income	< $6000/yr	16.3 (230)	15.3 (114)	17.3 (116)	0.60
	$6000 to $11,999/yr	51.6 (730)	53.2 (395)	49.9 (335)	
	$ 12,000 to $17,999/yr	21 (297)	20.3 (151)	21.8 (146)	
	$18,000+	11.1 (157)	11.2 (83)	11 (74)	
Number of adults and children living in household	1	54.6 (871)	52.2 (436)	57.2 (435)	0.008*
	2	17.1 (273)	18.2 (152)	15.9 (121)	
	3	12.3 (197)	12.2 (102)	12.5 (95)	
	4	8.8 (141)	10.3 (86)	7.2 (55)	
	5	4.5 (72)	5.3 (44)	3.7 (28)	
	6	1.7 (27)	0.8 (7)	2.6 (20)	
	7	0.4 (7)	0.6 (5)	0.3 (2)	
	8	0.4 (6)	0.2 (2)	0.5 (4)	
	9	0.1 (2)	0.2 (2)	0 (0)	
Food Assistance (Any of the following: WIC, SNAP (Food Stamps), Emergency Food Program, Summer Food Service Program, Senior Citizen Meal Program, Meals on Wheels)	Yes	82 (1305)	84.1 (702)	79.8 (603)	0.03*
	No	18 (286)	15.9 (133)	20.2 (153)	

*indicates statistical significance (*p* < 0.05)

The experimental groups were balanced at baseline on most demographic characteristics. However, the intervention group had slightly more participants in some of the younger age groups compared with the control group (*p* < .01); though the mean age was not different between groups. Also, the intervention group had slightly fewer participants living alone (52%) compared with the control group (57%, *p* < .01).

Process evaluation data indicated that over the course of the year-long intervention, an average of 23 markets were held at each site with a smaller average number of markets at the family sites (21.3 markets) than at the elderly sites (23.8 markets). Elderly sites also had a higher average number of customers and sales per market (average of 29 customers and $245 in sales) compared with the family sites (average of 9 customers and $82 in sales). For elderly sites, more of the sales were paid for with cash (43%) than with SNAP (37%), while at the family sites, the opposite was true (cash 33%, SNAP 50%). Less than 20% of participants used debit or credit cards. The top five selling F&V were bananas, cucumbers, red grapes, navel oranges and strawberries for the elderly sites and strawberries, bananas, navel oranges, red grapes and cilantro for the family sites.

At the 12-month survey (Table 2), more than half of the evaluation cohort participants (57%) reported that they had attended a few of the FTY markets; 18.6% attended some of the markets; while only 7.7% attended all or most of the markets; and 16.6% reported that they had not attended any of the markets. About half of the participants reported that FTY market prices (47%) and F&V quality (51%) was about the same as at local grocery stores; while 33% reported that the FTY prices and F&V quality (48%) was better than at grocery stores. The majority (74%) rated the FTY market F&V quality as very good or excellent and the F&V prices as very good or excellent (53%). The large majority (80%) of participants reported that the FTY markets always or often sold the F&V that they liked and that they purchased about the same amount of F&V at the FTY markets as they did at grocery stores. Most participants (65%) reported that no one else in their household shopped at the markets.

**Table 2:** Intervention Group Evaluation Cohort Participant Self-Reported F&V Market participation and Satisfaction at 12 months (*N* = 653)

	Frequency	Percent
Would you say you shopped at…?		
None of the markets	108	16.6
A few of the markets	373	57.2
Some of the markets	121	18.6
Most of the markets	26	4.0
All of the markets	24	3.7
Would you say the prices of the FV sold at the Fresh To You markets were….?		
Lower than grocery store prices	176	32.6
About the same as grocery store prices	251	46.5
Higher than grocery store prices	113	20.9
How would you rate the cost of the FV sold at the Fresh To You markets?		
Poor	7	1.3
Not so good	50	9.2
Good	201	37.0
Very good	148	27.3
Excellent	137	25.2
Would you say the quality of the FV sold at the Fresh To You markets was…?		
Worse than what is sold at grocery stores	8	1.5
About the same as what is sold at grocery stores	277	50.8
Better than what is sold at grocery stores	260	47.7
How would you rate the quality of FV sold at the Fresh To You markets?		
Poor	2	0.4
Not so good	15	2.8
Good	122	22.4
Very good	174	31.9
Excellent	232	42.6
How often did the Fresh To You markets sell the types of FV that you like to eat?		
Always	289	53.1
Often	146	26.8
Sometimes	91	16.7
Rarely	15	2.8
Never	3	0.6
Did anyone else in your household ever shop at the Fresh To You markets?		
Yes	178	35.3
No	327	64.8

Educational campaigns were provided to intervention and control groups with varied success. Table 3 details the self-reported participation of evaluation cohort participants in the various educational activities. A lower proportion of evaluation cohort participants in the comparison group reported attending the “Take 10” physical activity campaign (25%) and “Stress Less” campaigns (20%) compared with participants in the intervention group evaluation cohort who reported attending the “Just Add 2,” (46%) and, “Color Your Plate,” (38%) F&V campaigns. Of those who did not join campaigns, the highest reason reported by both groups was not hearing about the campaign (data not shown). Of those who did participate, about 75% of respondents reported that the campaigns were very helpful.

**Table 3:** Self-reported Participation of evaluation cohort participants in the educational intervention components. (6 Months *N* = 1354, 12 Months *N* = 1275)

Campaign								
	Control				Intervention			
	6 Months		12 Months		6 Months		12 Months	
	*Take 10*		*Stress Less*		*Just Add 2*		*Color Your Plate*	
	Frequency	%	Frequency	%	Frequency	%	Frequency	%
Did you join the campaign?								
Yes	159	24.6	122	19.9	317	46.5	244	38.1
No	487	75.4	491	80.1	365	53.5	396	61.9
How helpful was the campaign?								
Very helpful	121	76.6	98	81.0	230	72.6	190	78.2
Somewhat helpful	11	7.0	10	8.3	40	12.6	32	13.2
A little helpful	22	13.9	12	9.9	40	12.6	17	7.0
Not at all helpful	4	2.5	1	0.8	7	2.2	4	1.7
*Other Intervention Components for the Intervention Group Only.*								
	6 Months				12 Months			
	Frequency	Percent			Frequency		Percent	
How many of the recipes that you received in your LWVB binder did you try?								
All of them	54	7.9			70		10.8	
Most of them	106	15.5			105		16.2	
Some of them	323	47.2			306		47.1	
None of them	158	23.1			135		20.8	
I didn’t get any recipes	43	6.3			34		5.2	
How many of the monthly newsletters did you read?								
I didn’t get any newsletters	0	0			70		11.2	
None of them	0	0			63		10.1	
1	59	9.0			35		5.6	
2	103	15.7			92		14.7	
3	87	13.3			77		12.3	
4	58	8.8			65		10.4	
5	39	6.0			54		8.7	
6	138	21.0			33		5.3	
7	75	11.4			11		1.8	
8	97	14.8			13		2.1	
9					5		0.8	
10					28		4.49	
11					2		0.3	
12					76		12.2	
How helpful were the newsletters that you did read?								
Very helpful	319	66.2			340		69.3	
Somewhat helpful	105	21.8			96		19.6	
A little helpful	49	10.2			46		9.4	
Not at all helpful	9	1.9			9		1.8	
How many of the DVDs did you watch?								
All 3 of them	80	11			94		14.5	
Two of them	101	14.6			104		16.0	
One of them	129	18.7			116		17.9	
None of them	272	39.4			250		38.5	
I didn’t get any DVDs	109	15.8			85		13.1	
How helpful were the DVDs that you watched?								
Very helpful	210	67.7			234		75.0	
Somewhat helpful	61	19.7			44		14.1	
A little helpful	34	11.0			32		10.3	
Not at all helpful	5	1.6			2		0.6	
Did you attend any of the taste-testing?								
Yes	181	26.3			228		35.0	
No	508	73.7			424		65.0	
How much did the taste-testing event affect what you bought at the market?								
A lot	60	33.3			81		35.5	
A little	60	33.3			71		31.1	
Some	28	15.6			32		14.0	
Not at all	32	17.8			44		19.3	
Did anyone else in your household use any of the LW educational materials?								
Yes	132	26.0			165		32.8	
No	375	74.0			338		67.2	

Participation in other intervention components was also mixed. For recipes, 27% of intervention group cohort participants reported using all or most of the recipes, while 47% reported using some and 21% reported using none of the recipes. Of the 12 monthly newsletters, 62% reported reading 6 or fewer of the newsletters, but the majority of those that did read them found them very (69%) or somewhat (20%) helpful. For the DVDs, 48% watched at least one DVD, and those who did watch them, found them to be very (75%) or somewhat (14%) helpful. For the taste-testing events, 35% reported attending at least one event with 31% reporting that these events affected their purchasing a lot and 14% reporting that they affected their purchasing some. One-third of participants reported that others in their household used the LWVB intervention materials.

Mixed models (Table 4) examining the effect of time overall showed that there were significant changes in F&V intake over time favoring the intervention group, (*p* < .02). From baseline to 12 months, the intervention group increased total F&V intake by 0.44 cups with the control group decreasing intake by 0.08 cups. An increase in the frequency of F&V eating behaviors was also observed as demonstrated by increases in the F&V Habits Questionnaire score over time, with the intervention group increasing by 0.24 and the control group increasing by 0.14 from baseline to 12 months (*p* < .01).

**Table 4:** Daily Fruit and Vegetable Intake of cups/day at Baseline and 12-month follow-up (Baseline *N* = 1597, 12 Months *N* = 1275)^#^

Outcome measurement		Baseline Mean (SE)	12 Month FU Mean (SE)	*p*-value (group x time)^#^
Fruit (no juice)	Intervention	1.23 (0.04)	1.39 (0.06)	
	Control	1.35 (0.06)	1.31 (0.07)	
	differences	−0.12 (0.07)	0.08 (0.09)	0.056
Vegetable (no fries)	Intervention	2.04 (0.09)	2.32 (0.15)	
	Control	2.13 (0.12)	2.10 (0.06)	
	differences	−0.09 (0.15)	0.22 (0.16)	0.01*
FV combined	Intervention	3.25 (0.10)	3.69 (0.20)	
	Control	3.49 (0.17)	3.41 (0.09)	
	differences	−0.24 (0.19)^a^	0.28 (0.22)	0.015*
FVHQ	Intervention	3.30 (0.07)	3.54 (0.06)	
	Control	3.31 (0.07)	3.45 (0.06)	
	differences	−0.01 (0.10)	0.09 (0.08)	0.01*

All models were adjusted for age

*indicates statistical significance (*p* < 0.05)

^#^6 month data included in models but not shown in table

While the study was not originally powered to look at F&V changes by housing site type, we decided to examine the study results within elderly/disabled and housing sites because study staff noticed differences in enthusiasm for the intervention by site type. Table 5 shows the F&V changes for the elderly housing sites and family sites separately. Mixed models examining the effect of time overall showed that there were significant changes in F&V intake over time favoring the intervention group, (*p* < .008) in the elderly sites. From baseline to 12 months, the intervention group increased total F&V intake by 0.65 cups with the control group decreasing intake by 0.07 cups, *p* = .0111. This change was driven more by increases in vegetable intake (*p* < .008) than increases in fruit intake (*p* = 0.2). In the family sites, we did not observe any significant differences in F&V change over time by experimental group.

**Table 5:** Daily Fruit and Vegetable Intake of cups/day at Baseline and 12-month follow-up by site type up (Baseline *N* = 1597, 12 Months *N* = 1275)^#^

Outcome measurement		Baseline Mean (SE)	12 Month FU Mean (SE)	*p*-value (group x time)^#^
Elderly/Disabled Housing Sites Only				
Fruit (no juice)	Intervention	1.27 (0.05)	1.45 (0.09)	
	Control	1.34 (0.09)	1.36 (0.07)	
	differences	−0.13 (0.10)	0.09 (0.12)	0.20
Vegetable (no fries)	Intervention	2.17 (0.11)	2.58 (0.17)	
	Control	2.26 (0.17)	2.17 (0.08)	
	differences	−0.09 (0.20)	0.41 (0.19)	0.008*
F&V combined	Intervention	3.37 (0.12)	4.02 (0.27)	
	Control	3.61 (0.26)	3.54 (0.13)	
	differences	−0.25 (0.28)	0.49 (0.30)	0.011*
Family Housing Sites Only				
Fruit (no juice)	Intervention	1.26 (0.03)	1.29 (0.02)	
	Control	1.36 (0.05)	1.23 (0.12)	
	differences	−0.10 (0.06)	0.06 (0.13)	0.25
Vegetable (no fries)	Intervention	1.85 (0.09)	1.94 (0.10)	
	Control	1.97 (0.07)	2.02 (0.05)	
	differences	−0.12 (0.11)	−0.08 (0.11)	0.78
F&V combined	Intervention	3.08 (0.13)	3.21 (0.07)	
	Control	3.34 (0.09)	3.26 (0.10)	
	differences	−0.26 (0.16)	−0.06 (0.12)	0.35

All models were adjusted for age

*indicates statistical significance (*p* < 0.05)

^#^6 month data included in models but not shown in table

### Dose response analysis

To better understand the dose response effect of specific intervention components, use of each component by intervention site participants was examined in relation to F&V intake (Table 6). Greater use of the markets was associated with greater change in intake of F&V at 12 months. There was a clear dose response effect with F&V intake change increasing with each level of increased market attendance from no markets to all of the markets (*p* < .05). Individuals who reported attending all or most of the markets increased F&V intake by 2.1 cups and 0.86 cups, respectively compared with less than half cup increases for lower levels of market attendance.

**Table 6:** Change in F&V intake of cups/day at each level of market attendance and participation in educational intervention components at 12 months (*N* = 653)

Markets Attended						
Change In	All Mean (Std. Dev) (n)	Most Mean (Std. Dev) (n)	Some Mean (Std. Dev) (n)	Few Mean (Std. Dev) (n)	None Mean (Std. Dev) (n)	*P*-value (2 sided)
Fruit (no juice)	0.96 (1.98) (26)	0.52 (1.52) (23)	0.05 (1.64)(91)	0.14 (1.50)(400)	0.13 (1.48)(108)	0.07
Vegetables (no fries)	1.08 (2.55) (23)	0.32 (2.55) (22)	0.34 (1.93)(86)	0.20 (1.99)(382)	0.12 (1.71)(99)	0.30
Fruit+Veg (no fries, no juice)	2.08 (3.09) (23)	0.86 (3.30) (22)	0.38 (2.92)(86)	0.34 (2.69)(381)	0.24 (2.56)(99)	0.046*
Recipes Tried						
Variable		All Mean (Std. Dev) (n)	Most Mean (Std. Dev) (n)	Some Mean (Std. Dev) (n)	None /I didn’t get recipes Mean (Std. Dev) (n)	*P*-value (2 sided)
Fruit (no juice)		0.33 (1.75)(69)	0.22 (1.61)(105)	0.13 (1.54)(306)	0.14 (1.45)(166)	0.79
Vegetables (no fries)		0.49 (3.13)(64)	0.70 (2.01)(98)	0.19 (1.85)(294)	−0.05 (1.54)(155)	0.02*
Fruit+Veg (no fries, no juice)		0.89 (4.00)(64)	0.98 (2.50)(98)	0.30 (2.71)(294)	0.08 (2.28)(154)	0.03*
Newsletter						
	Read at least Half			Did not read at least half		*P*-value (2 sided)
Fruit (no juice)	0.21 (1.66)(287)			0.14 (1.48)(333)		0.57
Vegetables (no fries)	0.24 (2.19)(274)			0.22 (1.83)(314)		0.93
Fruit+Veg (no fries, no juice)	0.43 (3.02)(274)			0.38 (2.55)(313)		0.84
DVDs						
	Watched at least one DVD			Watched no DVDs		*P*-value (2 sided)
Fruit (no juice)	0.19 (1.57)(313)			0.15 (1.53)(332)		0.71
Vegetables (no fries)	0.47 (2.20)(302)			0.02 (1.74)(308)		0.005*
Fruit+Veg (no fries, no juice)	0.69 (2.89)(302)			0.15 (2.60)(307)		0.015*
Taste Tests						
	Attended any taste tests			Attended no taste tests		*P*-value (2 sided)
Fruit (no juice)	0.38 (1.71)(228)			0.06 (1.44)(420)		0.012*
Vegetables (no fries)	0.47 (2.26)(216)			0.11 (1.82)(396)		0.032*
Fruit+Veg (no fries, no juice)	0.84 (3.18)(216)			0.18 (2.46)(395)		0.004*
Campaign Participation						
	Attended any of the campaign events			Attended no campaign events		*P*-value (2 sided)
Fruit (no juice)	0.28 (1.62)(244)			0.09 (1.49)(393)		0.13
Vegetables (no fries)	0.23 (2.16)(231)			0.20 (1.76)(372)		0.84
Fruit+Veg (no fries, no juice)	0.51 (2.94)(231)			0.30 (2.50)(371)		0.34

*indicates statistical significance (*p* < 0.05)

Reading of newsletters or attending any campaign events was not associated with any change in F&V consumption. However, watching of the DVDs, even one compared with none at all, was associated with a 0.47 cup increase in consumption of vegetables (*p* < .005) and 0.69 cup increase in F&V combined (*p* < .02). In addition, those who tried most or all of the recipes had higher increases in vegetable and F&V intake than those who used few or no recipes (*p* = 02 and 0.03, respectively) and those who attended at least one taste test had higher increases in fruit, vegetable and F&V intake than those who attended no taste tests (*P* = 0.01, 0.03 and 0.004, respectively).

Responses to the open-ended questions revealed additional information about participants’ reactions to LWVB. When intervention group participants reported what they liked most about LWVB, the most common responses were related to the information provided (e.g. “it educates you, makes you change your eating habits”). The top specific categories were related to convenience/accessibility (e.g. “Didn’t have to lug 20 pounds from the grocery store”), recipes, education about F&V (e.g. “the information on what F&V you should be eating”), and the quality of the F&V (e.g. “very fresh”). When asked what they had learned from LWVB, participants mentioned the importance of eating more F&V. They also talked about how to eat healthier in general and how to prepare F&V (including using new recipes). They also discussed the importance of eating a variety of F&V. When asked how the LWVB project could be improved, most respondents said that it didn’t need improvement. Others cited lower prices, more education and recipes, to continue to bring LWVB to them, and to offer different market times. Additionally, participants suggested that we improve our reach to others in the community with more and better advertising.

## Discussion

‘Live Well, Viva Bien’ (LWVB) is the first cluster, randomized controlled trial to demonstrate the efficacy of year-round F&V markets at improving F&V intake for low-income adults. Overall, the LWVB intervention increased F&V intake more than the control group by approximately 0.52 cups and there was a dose-response effect demonstrating that greater market attendance resulted in greater F&V intake. Non-experimental studies have shown that seasonal farmers’ markets selling local produce can increase F&V intake of participants; but these studies used cross-sectional or one-group, repeated-measures designs [33–37], not randomized controlled trials. Moreover, these studies only measured F&V change of market participants. Several other studies have examined the efficacy of educational programs and/or monetary vouchers for F&V at farmer’s markets; but did not study the efficacy of the markets themselves [38, 39].

The results of the LWVB study provide strong evidence in support of the hundreds of mobile F&V markets around the country, which may facilitate procurement of funding that requires an evidence-base. For example, this study’s demonstration that mobile markets can increase F&V intake may allow such markets to be eligible for community benefit funds from local nonprofit hospitals that seek federal tax exempt status [40–43]. As of December 2014, federal rules allowed such hospitals to consider funding programs that “…prevent illness, to ensure adequate nutrition, or to address social, behavioral, and environmental factors that influence health in the community” [42] as long as the programs they fund are evidence-based.

The LVWB study found that the elderly/disabled housing sites had greater changes in F&V intake in comparison to the family sites. This finding is likely related to higher involvement in the markets and educational interventions by the elderly/disabled site residents compared to the family site residents, and that elderly individuals, in general, are likely to be more health conscious than younger individuals. At the elderly/disabled sites, the markets were held indoors so participants only needed to take the elevator downstairs to shop, while at family sites, the markets were held outside or in non-adjacent buildings, which required residents to leave their apartments and walk down the street to shop. Another explanation may pertain to elderly/disabled subpopulations being particularly vulnerable to barriers in food access. In other words, they may be hypersensitive to food environment changes made in close proximity to their homes because of physical limitations or inability to drive to a grocery store [44]. Future studies should consider how to maximize such programs for elderly and disabled individuals as well as how to create programs that might be more attractive to younger families.

The dose response data also indicate that higher market participation was associated with greater increases in F&V intake. This is an important finding, which highlights the significance of regular market attendance and reach. A systematic review by Freedman and collaborators identified several factors influencing farmer’s market use that are likely applicable to mobile market participation, including economic facilitators/barriers, service delivery, spatial/temporal, social, and personal factors [45]. More research is needed to determine how to modify future markets to encourage increased participation and greater reach. We conducted group concept mapping to examine factors that may have affected FTY market participation and will publish the results in a subsequent publication.

The results of LWVB also inform the field about educational programming in conjunction with F&V market programs. LWVB process evaluation data indicate that participation in most of the educational intervention components (campaigns, newsletters, DVDs, recipes and taste tests) was not particularly high. Of these components, watching the DVDs, attending taste-testing events and using recipes were associated with increased F&V intake. The LWVB DVDs mostly featured cooking segments, which appeared to be of interest to study participants as well as the recipes corresponding to these cooking segments. These findings provide important implications for future interventions especially with low-income, less literate audiences. An audiovisual intervention may be the best approach for reaching such groups. Future interventions should study how to repackage such interventions to reach more F&V market participants. For example, should they be distributed as DVDs at markets or would it be more effective to send video clips and/or recipes through email or text links? Would it be helpful to provide scan codes at point-of-purchase that would then send links to videos and/or recipes via email or text? The reach, feasibility, acceptability and efficacy of such interventions could be examined in future studies.

Alternatively, in order to increase participation in the educational components, future interventions might consider embedding the nutrition education component as a requirement for participants to receive discount produce or financial benefits. This strategy was employed in the Health Bucks study, a randomized control trial, which found that coupling an hour-long, group nutrition education program with a small, economic incentive ($6 coupon) was effective at increasing F&V purchasing and consumption among overweight patients with diabetes [46].

Taste-testing events may also be important at mobile markets as a draw to increase attendance at the markets and to provide opportunities for consumers to try F&V and receive recipes for preparing them in new and different ways. Cost-efficient ways to implement taste-testing events at mobile markets could include working with SNAP-Ed, the Expanded Food and Nutrition Education Program (EFNEP) or local culinary schools that could provide such events as a community service.

Discount, mobile produce markets like those in the LWVB study have the opportunity to not only increase F&V consumption; but also to increase food security because the F&V prices are lower than supermarket prices. However, while LWVB markets had lower prices than supermarkets, they still appeared to be too high for participants to purchase large amounts of F&V. Sales averaged only $8.55 per person and some participants reported that FTY market prices were still too high. As this was a research efficacy study it did not focus on, nor did it evaluate the cost or profitability of the markets. The LWVB markets required extra staff and a POS system for evaluation purposes and thus could not estimate costs for markets in a non-research situation. Most mobile F&V markets operating in food deserts or low income neighborhoods are non-profits that require donations and/or grant funds to operate [47]. Future studies should look at costs and cost-effectiveness of mobile F&V markets and market plus education interventions like LWVB and determine ways in which mobile markets could be self-sustaining. Potential approaches to reducing per market cost could be using housing site residents or other volunteers as market staff, or having students work at the markets for their community service requirements. Profitability could possibly be increased by increasing reach and/or by selling other items at the mobile market besides produce.

Financial incentives may be important for low-income resident participation in farmers’ markets and mobile F&V markets. A study by Freedman and colleagues points to the effectiveness of monetary incentives and SNAP-EBT and WIC acceptance at increasing the use of farmers’ markets among WIC recipients [48]. While LWVB markets did accept SNAP-EBT, they could not accept WIC because FTY was not an approved WIC vendor or a “farmers’ market” selling only local F&V. The WIC Farmers’ Market Nutrition Program (FMNP) [49] was established in 1992 to provide fresh, unprepared, locally grown F&V to WIC participants, and to expand the awareness, use of, and sales at farmers’ markets. Women, infants (over 4 months old) and children who have been certified to receive WIC program benefits or who are on a waiting list for WIC certification are eligible to participate in the WIC FMNP. In RI, the farmers’ market vouchers are valid for one season, from June through October. Generally, such vouchers have not been fully utilized with redemption rates in RI averaging less than 40% (personal communication, RI Department of Health). Barriers have included high F&V prices, limited seasonal availability and inconvenient locations of some farmers’ markets. If it were possible for year-round mobile markets, like FTY, that sell F&V from local farms as well as culturally desired, non-local produce, to accept WIC, low-income families could have year-round access to affordable, fresh produce at convenient locations. Allowing WIC FMNP coupons to be accepted at mobile markets as well as at farmers’ markets would enable more coupons to be redeemed, thereby benefitting local farmers as well as low-income families.

Financial incentives such as those offered by the United States Department of Agriculture’s Food Insecurity Nutrition Incentive (FINI) program may also help to increase the fresh F&V purchases of low-income families. The FINI program enables SNAP recipients to receive coupons that increase the monetary value of their SNAP dollars for the F&V they buy at produce markets [50, 51]. For example some programs provide a coupon that doubles the value of SNAP benefits, e.g. buy $15 of F&V and get a matching $15 coupon that can be used to buy more F&V; while other programs provide a coupon worth 2/5 or 3/5 of the amount of F&V purchased with SNAP benefits. Ongoing studies are evaluating the effectiveness of these programs; however, further research on the relationship between pricing, financial incentives and F&V intake in low-income families is needed.

Moreover, in LWVB we found that market participation dropped off substantially after the first 2 weeks of the month when residents’ SNAP benefits ran out. This phenomenon has been shown to be an issue locally and nationally [52, 53] and has been related to increased food insecurity, increased hospital admissions, lower student test scores and increased disciplinary events among school-aged children in later weeks of the month [54]. Future research should look at ways to circumvent this problem, for example allowing FINI coupons to be used by SNAP recipients to purchase F&V at produce markets even after their EBT benefits have run out for the month or providing SNAP-EBT benefits more frequently, e.g., weekly or biweekly, rather than just once per month.

Another approach that could be used to decrease the cost and increase the purchase and consumption of F&V would be to have medical professionals write prescriptions for F&V that would then be covered by insurance and redeemed at the markets. This approach has been used and tested by Wholesome Wave in various sites across the county. They have also published a detailed toolkit for other organizations interested in implementing such an approach [55]. Further, recent empirical studies of F&V prescription programs indicate positive impacts on healthy food access, food insecurity, perceived health, hemoglobin levels among diabetes as well as BMI status [56–59].

Despite the positive results of the LWVB intervention on F&V intake, several study limitations need to be noted. The study sample included mostly (73%) women with few men participating. The preponderance of female participants can be attributed mostly to the overall higher percentage of women living in the housing complexes, particularly in the family sites. In addition, one of the evaluation cohort eligibility requirements was that participants needed to shop for household food at least half of the time. While men and woman are sharing food shopping responsibilities more than in the past, women are still more likely to have primary responsibility for food shopping [60]. F&V intake was measured via self-report (although validated tools were used and a control group allowed for comparisons between exposures with similar measures). Market attendance at the FTY markets was not consistently high at some of the housing sites, particularly the family sites, and participation in many of the educational intervention components was also relatively low. The evaluation was conducted on all participants in the evaluation cohort, not just on intervention participants; so the overall analysis is conservative with respect to the effect of the intervention. However, the dose response analysis demonstrates the effectiveness of the intervention on those who participated. The LWVB study could not completely differentiate the effect of the market intervention vs. the educational intervention, so future studies should be designed to separate these effects. Strengths of the study included the cluster, randomized controlled trial study design, the formative research used to design the interventions, the community collaborations, diversity of the study population, the use of validated measures, the extensive process evaluation and dose response evaluation. Additionally, the large number and diversity of housing sites and participants allows for better generalizability of the results.

## Conclusions

The findings from the LWVB study make a substantial contribution to the field by providing important scientific evidence regarding the efficacy of mobile produce market programs. Further, our results more broadly support investment in environmental changes to alleviate disparities in F&V consumption and diet-related health inequities. Now that we know that mobile market programs can be effective at increasing F&V intake of low-income consumers, future implementation and dissemination research is needed regarding how to increase reach and participation in such programs, as well as how to deliver educational programming in effective and feasible ways. Other adaptations that might be considered to improve future mobile markets include: expanding market inventory to include often-requested staple, non-produce food items; working with state WIC programs to allow WIC coupon redemption at mobile markets; finding better locations for reaching low-income families; partnering with community stakeholders bringing mobile events to underserved neighborhoods, e.g., blood drives, SNAP enrollment events, health fairs, summer food programs, etc.; including incentive programs, e.g., “double up” SNAP benefits; and engaging students and/or other volunteers as market/education staff. Future studies should measure not only F&V intake, but also the impact of F&V market programs on total diet, food insecurity and/or health outcomes.

## Appendix

**Table 7:** Intraclass Correlation Coefficients (ICC) from 3-level hierarchical model (Baseline *N* = 1597, 6 Months *N* = 1354, 12 Months *N* = 1275)

	Across time within residents of the same site	Between two residents of the same site
Fruit	0.370	0.003
Vegetables	0.368	0.018
Fruit and Vegetables	0.423	0.014
FVHQ	0.643	0.053

## Abbreviations

DVD: Digital video disk
EBT: Electronic benefits transfer
F&V: Fruits and vegetables
FTY: Fresh to you
LWVB: Live Well, Viva Bien
NCI: National Cancer Institute
POS: Point-of-sale
SCT: Social cognitive theory
SES: Socioeconomic status
SNAP: Supplemental nutrition assistance program
